# Clinic friendly estimation of muscle composition: Preoperative linear segmentation shows overall survival correlated with muscle mass in patients with nonmetastatic renal cell carcinoma

**DOI:** 10.3389/fonc.2022.1068357

**Published:** 2022-11-25

**Authors:** Benjamin N. Schmeusser, Eric Midenberg, Arnold R. Palacios, Nikhil Vettikattu, Dattatraya H. Patil, Alexandra Medline, Michelle Higgins, Manuel Armas-Phan, Reza Nabavizadeh, Shreyas S. Joshi, Vikram M. Narayan, Sarah P. Psutka, Kenneth Ogan, Mehmet A. Bilen, Viraj A. Master

**Affiliations:** ^1^ Department of Urology, Emory University School of Medicine, Atlanta, GA, United States; ^2^ Department of Urology, University of Louisville, Louisville, KY, United States; ^3^ Department of Urology, Creighton University, Omaha, NE, United States; ^4^ Department of Internal Medicine, Beth Israel Deaconess Medical Center, Boston, MA, United States; ^5^ Brady Urological Institute, Johns Hopkins Hospital, Baltimore, MD, United States; ^6^ Department of Urology, Mayo Clinic, Rochester MN, United States; ^7^ Department of Urology, University of Washington, Seattle, WA, United States; ^8^ Fred Hutchinson Cancer Center, University of Washington, Seattle, WA, United States; ^9^ Department of Hematology and Medical Oncology, Emory University School of Medicine, Atlanta, GA, United States

**Keywords:** sarcopenia, muscle mass, muscle composition, nephrectomy, renal cell carcinoma, linear segmentation

## Abstract

**Purpose:**

Sarcopenia is associated with decreased survival and increased complications in patients with renal cell carcinoma. Readily identifying patients with low muscle composition that may experience worse outcomes or would benefit from preoperative intervention is of clinical interest. Traditional body composition analysis methods are resource intensive; therefore, linear segmentation with routine imaging has been proposed as a clinically practical alternative. This study assesses linear segmentation’s prognostic utility in nonmetastatic renal cell carcinoma.

**Materials and Methods:**

A single institution retrospective analysis of patients that underwent nephrectomy for nonmetastatic renal cell carcinoma from 2005-2021 was conducted. Linear segmentation of the bilateral psoas/paraspinal muscles was completed on preoperative imaging. Total muscle area and total muscle index associations with overall survival were determined by multivariable analysis.

**Results:**

532 (388 clear cell) patients were analyzed, with median (IQR) total muscle index of 28.6cm^2^/m^2^ (25.8-32.5) for women and 33.3cm^2^/m^2^ (29.1-36.9) for men. Low total muscle index was associated with decreased survival (HR=1.96, 95% CI 1.32-2.90, p<0.001). Graded increases in total muscle index were associated with better survival (HR=0.95, 95% CI 0.92-0.99, p=0.006).

**Conclusions:**

Linear segmentation, a clinically feasible technique to assess muscle composition, has prognostic utility in patients with localized renal cell carcinoma, allowing for incorporation of muscle composition analysis into clinical decision-making. Muscle mass determined by linear segmentation was associated with overall survival in patients with nonmetastatic renal cell carcinoma.

## Introduction

In 2021, there were 79,000 newly diagnosed cases of kidney cancer with an estimated 14,920 attributed deaths (1). Nephrectomy with the goal of curative resection remains the gold standard in the management of localized renal cell carcinoma (RCC) (2). However, there are limited prognostic tools that allow clinicians to counsel patients on the perioperative mortality and morbidity risks associated with nephrectomy (3) Body composition analysis has the potential to risk stratify and prognosticate patients undergoing surgery for RCC.

Sarcopenia, defined as skeletal muscle mass paucity associated with reduced function (4), is associated with decreased survival, postoperative complications, and systemic therapy toxicity in nearly all solid organ malignancies (5–10). Routine imaging obtained during the preoperative workup of RCC, such as computed tomography (CT) or magnetic resonance imaging (MRI), can be utilized to accurately quantify body composition parameters (11–13). However, traditional body segmentation methods are time intensiv, require training, and involve the use of a specialized software which is expensive and not widely available, limiting the widespread adoption of integrating these measurements in clinical practice.

Linear segmentation, first proposed by Avrutin et al. (11), is less time intensive and would be well suited for clinical practice. It is accomplished by using a digital ruler to measure the length and width of the psoas and paraspinal muscle groups at the level of the third lumbar vertebra (L3) on axial imaging. This method was found to correlate well with traditional body segmentation methods among a patient cohort of intensive care unit patients (11, 14). Later, Feliciano et al. validated this method in a large study of 807 nonmetastatic colorectal cancer patients undergoing surgical resection, with results supporting the linear segmentation method in predicting total skeletal muscle mass. Importantly, this study also demonstrated utility in predicting survival outcomes (15).

The prognostic utility of linear segmentation has been demonstrated in colon cancer patients (15), but there remains a paucity of literature regarding its use in other cancer populations, including renal cell carcinoma. This study aims to examine the ability of preoperative linear segmentation to predict overall survival in patients undergoing nephrectomy for nonmetastatic RCC.

## Methods

### Patient demographics

The study received approval by the Institutional Review Board (IRB00055316). This is a retrospective cohort analysis of a prospectively maintained database examining patients with nonmetastatic RCC who underwent partial or radical nephrectomy between 2005-2021 at a single tertiary referral center. Patients with a histologically confirmed diagnosis of nonmetastatic RCC of any histology with a digital preoperative CT or MRI of the chest, abdomen, and pelvis within 60 days before surgery were included in the study. Preoperative patient-specific data including age, gender, race, body mass index (BMI; kg/m^2^), and Eastern Cooperative Oncology Group (ECOG) score were included as covariates. Postoperative tumor data including TNM staging, Fuhrman grade, and tumor size, as determined by the longest tumor diameter recorded in the pathology report, were included in the analysis. The 8^th^ edition of AJCC staging system for renal tumor classification was used for pathologic staging (16).

### Linear segmentation

**Figure 1:**
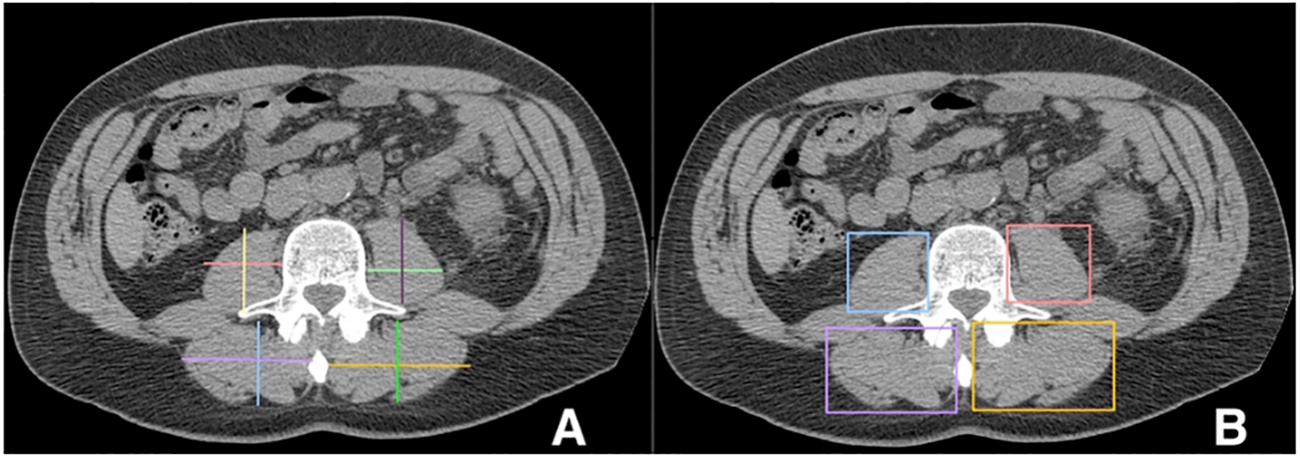
Linear measurements of the Lumbar Psoas and Paraspinal Musculature Using the Digital Ruler Tool **(A)** and the Box Measurement Tool **(B)** https://onlinelibrary.wiley.com/doi/full/10.1002/rco2.66.

**Figure 2:**
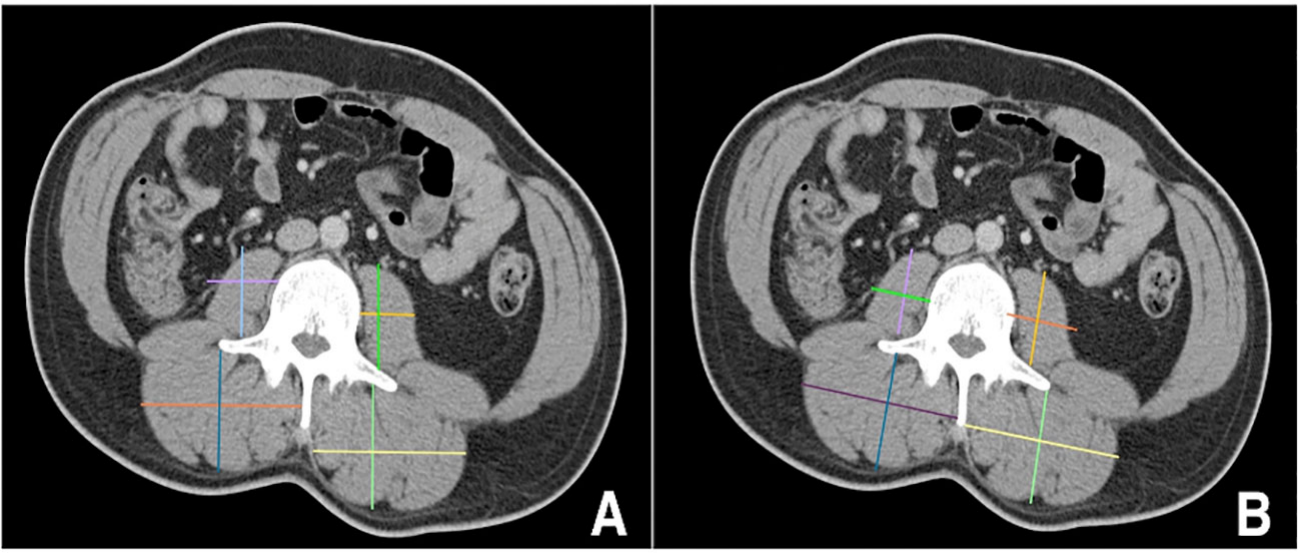
Incorrect **(A)** versus correct **(B)** orientation for obtaining linear measurements in patients mispositioned during their imaging scans https://onlinelibrary.wiley.com/doi/full/10.1002/rco2.66.

Linear segmentation was performed on preoperative axial imaging studies segmented at the mid-level of the third lumbar vertebrae, as successfully conducted in previous studies (14, 15, 17, 18). CT or MRI images were used due to reported agreement with both traditional skeletal muscle mapping and linear measures (18, 19). The skeletal muscle at the L3 vertebral level is examined due to its correlation with total skeletal muscle composition and its functional roles (11, 14, 20). Linear segmentation was completed by trained personnel following training requiring <5% interobserver variability and high intraobserver reliability as measured by intraclassical correlation, consistent with previous studies (19). Training is minimal, necessitating only location of the L3 level, recognition of psoas and paraspinal muscle boundaries, and comfortability with traditional radiology measuring tools. Each researcher was blinded to patient history and outcomes. Using Horos, a free, open-source medical image viewer (www.horosproject.org), the length and width of the individual psoas and paraspinal muscles at their longest and widest points were bilaterally measured. This was performed by orienting the vertical and horizontal digital ruler tool at an intersecting angle of approximately 90°, or by using the rectangular tool function (box method), which measures the same dimensions and ensures the 90° angle is met (**Figure 1**). In this study, the box method was primarily used. To account for potential inaccuracies in linear segmentation measurements in patients not perfectly oriented during their imaging study, psoas and paraspinal measurement were obtained at their longest and widest points in their vertical and horizontal orientation (**Figure 2**). Individual psoas and paraspinal muscle areas were calculated in cm^2^ by multiplying the length and width; total muscle area was calculated by aggregating the area of all four muscle groups. The total muscle index was calculated by dividing the total muscle area by height in m^2^.

A brief, real-time example of the linear measurements with the box method as described can be viewed in **Supplemental Video 1**, demonstrating the ability to complete these measurements in around one minute or less. For a more thorough explanation and demonstration of both total muscle area mapping and linear measurements, please refer to the video publication by Steele et al. (14)

### Statistical analysis

The primary exposure was total muscle index defined as both a binary and a continuous variable. The median value of the total muscle index stratified by sex was used as a cutoff value to delineate high or low skeletal muscle mass in respective sex. Our primary outcome was overall survival (OS) defined as the time from surgery to death from any cause or day of last follow-up recorded in patient charts.

Patient and clinical characteristics were described with a generalized chi-square test or Fisher’s exact test for categorical variables and a Wilcoxon rank-sum test for continuous variables. The prognostic value of the total muscle index stratified by sex above or below the median level was analyzed using the Kaplan-Meier method. Multivariable Cox proportional hazards regression models were fit using total muscle index, age at time of surgery, sex, race, BMI, and ECOG status as *a priori* selected variables. After collinearity and interaction assessment T-stage, N-stage, Furhman grade, clear cell histology, and necrosis were included in the model. Harrell’s concordance statistic estimate (c-index) was calculated for each model. Analyses were conducted on the full and clear cell RCC (ccRCC) cohort separately. A non-clear cell RCC cohort was not analyzed due to a varying number of many histological subtypes. All statistical tests were two-sided with type I error set at 0.05. All analyses were performed using SAS version 9.4 (Cary, NC, USA).

## Results

The characteristics of the cohort are summarized in **Table 1**. The full cohort consisted of 532 patients. Most patients were male (n=351 [66.0%]). Median (IQR) age and BMI at time of surgery was 61 years (19.5-91) and 29 kg/m^2^ (12.5-75), respectively. In total, 42.1%, 15.6%, 38.5%, and 3.8% of patients had T1, T2, T3, & T4 disease, respectively. The median number of days from preoperative scan to surgery day was 27 (0-60). The median (IQR) total muscle area was 77.9 (69.8-88.7) for women and 107.9 (93.2-118.3) for men. The median (IQR) total muscle index was 28.6 (25.8-32.5) for women and 33.3 (29.1-36.9) for men. To assess for potential bias introduced by varying histologies, a sensitivity analysis was performed evaluating muscle measures and associations with survival in patients with ccRCC (n=388) only, which had similar overall characteristics.

**Table 1:** Patient demographics for Localized RCC cohort.

	Full Cohort	ccRCC
Covariate	No. (%) (n=532)	No. (%) (n=388)
Age at surgery*	60.5 (19.5-91)	61.3 (19.5-89.9)
**Sex**		
Male	351 (66.0)	257 (66.2)
**Race**		
White	357 (67.1)	287 (74)
Black	143 (26.9)	74 (19.1)
Other	19 (3.6)	16 (4.1)
Unknown	13 (2.4)	11 (2.8)
**ECOG Status**		
ECOG ≥ 1	84 (15.8)	51 (13.1)
BMI*	29 (12.5-75)	29.2 (15.7-75)
**Obesity (≥ 30 kg/m2)**	233 (43.8)	179 (46.1)
**Type of Nephrectomy**		
Radical	374 (70.3)	268 (69.4)
**Preoperative Muscle**		
Total muscle area (cm2)**		
Women	77.9 (69.8-88.7)	77.5 (66.0-89.3)
Men	107.9 (93.2-118.3)	106.4 (91.9-117.7)
Total muscle index**		
Women	28.6 (25.8-32.5)	28.4 (25.4-33.2)
Men	33.3 (29.1-36.9)	33.0 (29.0-36.7)
Above Total Muscle Index Median	265 (50.2)	181 (46.6)
Below Total Muscle Index Median	267 (49.8)	207 (53.4)
Days preoperative scan to surgery*	27 (0-60)	25 (0-60)
**Fuhrman Grade**		
Grade 1-2	190 (36.7)	158 (41)
Grade 3-4	328 (63.3)	227 (59)
**pT-Stage**		
T1	224 (42.1)	171 (44.1)
T2	83 (15.6)	37 (9.5)
T3	205 (38.5)	168 (43.3)
T4	20 (3.8)	12 (3.1)
**Pathological N-Stage**		
N0	501 (94.2)	371 (95.6)

*median (min-max), **Median (IQR). Abbreviations: Total Muscle Area (TMA), Total Muscle Index (TMI)=[TMA]/height (m2), Eastern Cooperative Oncology Group (ECOG), Body Mass Index (BMI), Total muscle Area (TMA), Clear Cell Renal Cell Carcinoma (ccRCC).

**Figure 3:**
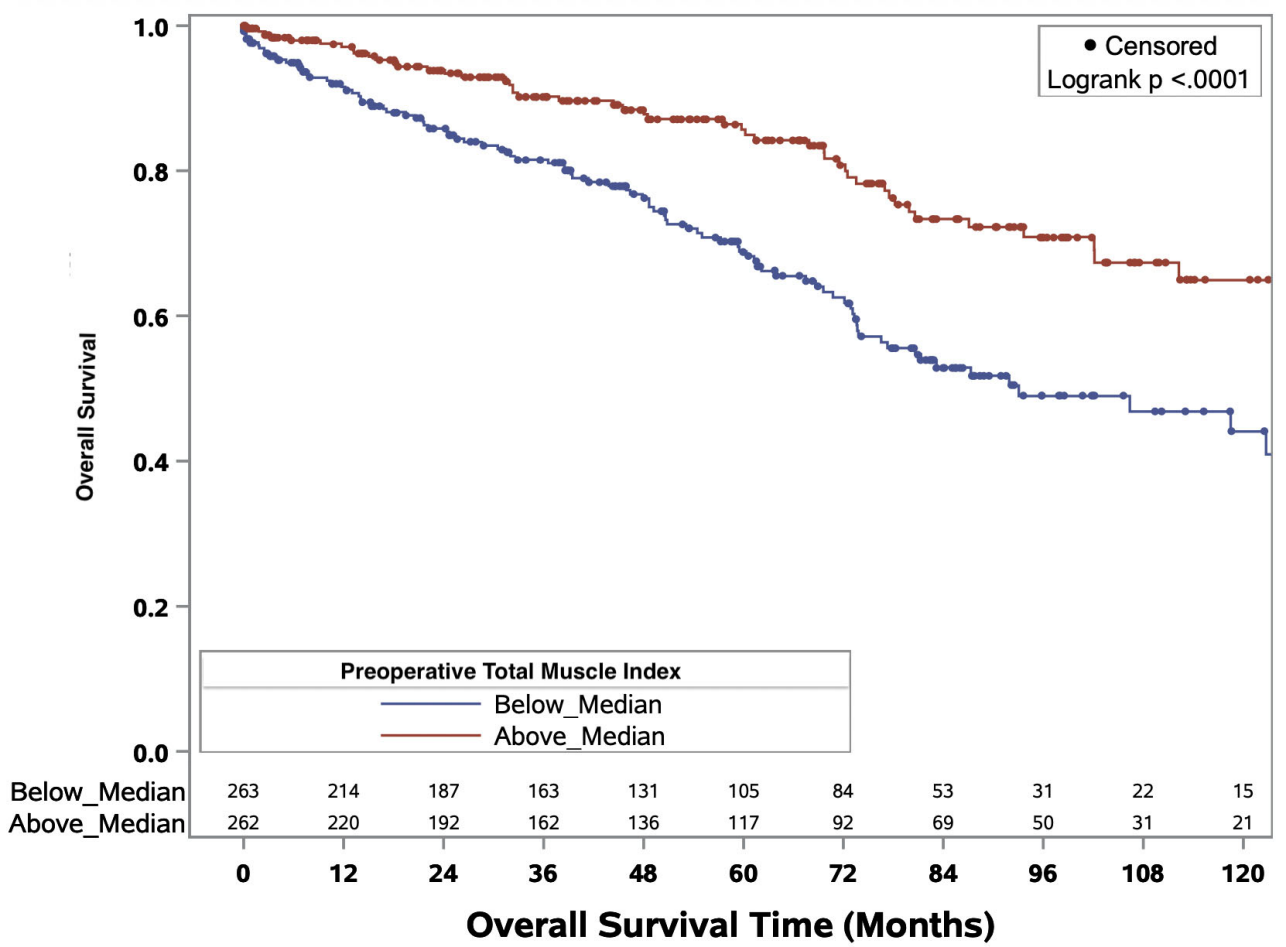
Kaplan-Meier curves evaluating median overall survival times in patients above or below median preoperative total muscle index for all RCC patients of any histology.

**Figure 4:**
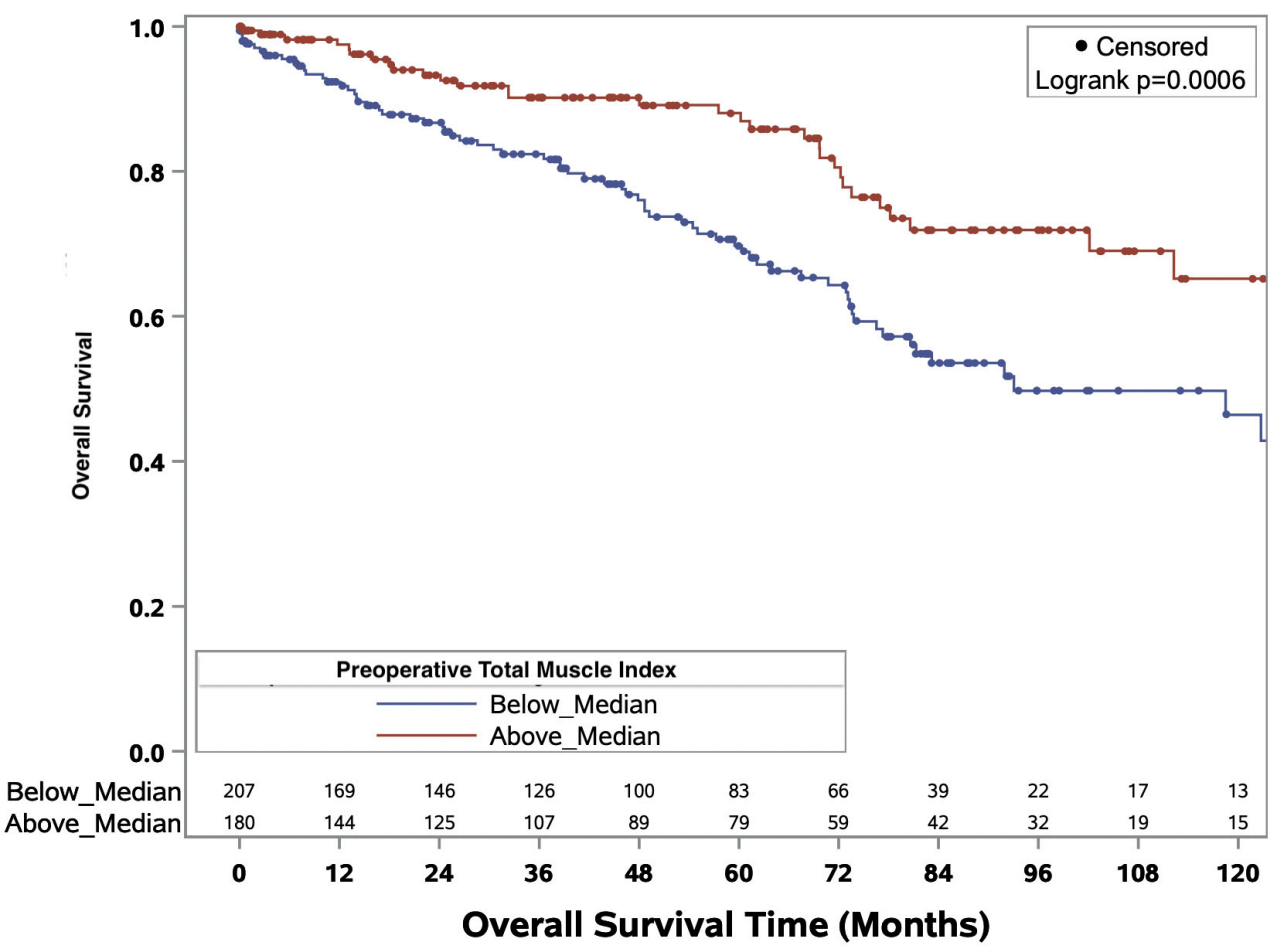
Kaplan-Meier curves evaluating median overall survival times in patients above or below median preoperative total muscle index for patients with clear cell renal cell carcinoma.

The Kaplan-Meier curves delineating the association between binary total muscle index and median overall survival times for the full cohort and ccRCC only cohort are illustrated in **Figures 3** and **Figure 4**, respectively. In the full cohort, median OS times were significantly decreased in patients with muscle index below the median level (p<0.0001; **Figure 3**). Similarly, in the ccRCC only cohort, preoperative muscle index above the median was associated with significantly improved overall survival (p=0.0006; **Figure 4**).

The median (IQR) postoperative follow-up time in months was 48.6 (*IQR=*20.5-82.4) for the full cohort and 46.4 (17.5-80.6) for the ccRCC cohort. The total number of deaths for the full and ccRCC cohorts were 147 and 105, respectively. Univariate Cox regression analyses were completed on both full and ccRCC only cohorts. In the full cohort, low total muscle index (binary variable), decreasing total muscle index (continuous variable), decreasing muscle area; age ≥60 years old; ECOG ≥1; Fuhrman grade; and T3,T4,and N1 disease were significantly associated with decreased OS. In the ccRCC cohort alone, below median total muscle index (binary variable), decreasing total muscle index (continuous variable), decreasing muscle area, age ≥60 years old, Fuhrman grade; and T3,T4,and N1 disease were significantly associated with decreased OS. No significant differences in OS on univariable analysis were observed between men and women in either cohort.

Results from multivariate Cox proportional hazards regression analysis with total muscle index as a binary variable (below or above median) are displayed in **Table 2**. Notably, low total muscle index was significantly associated with decreased overall survival in the full cohort (HR=1.96, 95% CI 1.32-2.90, p<0.001) and in the ccRCC cohort (HR=1.78, 95% CI 1.08-2.75, p=0.022). In addition to decreased total muscle index, age ≥60 years old was significantly associated with decreased OS in both the full and ccRCC only cohort. T3 disease was significantly associated with decreased OS in the full cohort only.

**Table 2:** Multivariable model summary of COX hazard overall survival for binary preoperative linear muscle index.

	Full Cohort		ccRCC	
Covariate	Hazard Ratio (95% CI)	HR P-value	Hazard Ratio (95% CI)	HR P-value
PreOp Total Muscle Index*				
Below Median	1.96 (1.32-2.90)	**<0.001**	1.72 (1.08-2.75)	**0.022**
**Age 60+**	1.59 (1.09-2.30)	**0.015**	1.62 (1.04-2.53)	**0.033**
**Gender**				
Male	1.13 (0.77-1.66)	0.54	1.19 (0.75-1.87)	0.459
**Race**				
Black	1.53 (1.00-2.36)	0.052	1.52 (0.87-2.63)	0.139
**Obesity (≥ 30 kg/m2)**	1.31 (0.91-1.89)	0.149	1.09 (0.71-1.68)	0.691
**ECOG**				
≥ 1	1.40 (0.93-2.10)	0.111	1.41 (0.85-2.34)	0.180
**Nephrectomy Type**				
Partial	0.65 (0.36-1.18)	0.155	0.56 (0.25-1.26)	0.159
**pT-Stage**				
T1	Ref	–	Ref	–
T2	0.92 (0.43-1.96)	0.824	0.78 (0.27-2.25)	0.650
T3	2.03 (1.07-3.86)	**0.03**	1.81 (0.85-3.86)	0.124
T4	2.71 (0.95-7.76)	0.063	2.91 (0.78-10.79)	0.110
**Pathologic N-Stage**				
N1	1.76 (0.97-3.21)	0.064	1.55 (0.68-3.53)	0.298
**Fuhrman Grade**				
G3-G4	1.14 (0.73-1.78)	0.554	0.90 (0.54-1.50)	0.675
**ccRCC**	0.98 (0.62-1.54)	0.928	–	–

*[TMA]/height (m2). Clear Cell Renal Carcinoma (ccRCC), Stage, Size, Grade, Necrosis (SSIGN), Eastern Cooperative Oncology Group (ECOG). C-index=0.7333 (full), 0.7246 (ccRCC). Bolded P-Value indicates clinical significance with p<0.05.

The association of muscle index as a continuous variable with OS was examined as well, with **Table 3** displaying the results from multivariable Cox proportional hazards regression analysis. A graded increase in total muscle index was significantly associated with better OS for the full cohort (HR=0.95, 95% CI 0.92-0.99, p=0.006) and ccRCC cohort (HR=0.95, 95% CI 0.91-0.99, p=0.016). T3 disease and age ≥60 years old were significantly associated with decreased overall survival in both the full cohorts as well.

**Table 3:** Multivariable model summary of COX hazard overall survival for continuous preoperative linear muscle index.

	Full Cohort		ccRCC	
Covariate	Hazard Ratio (95% Cl)	HR P-value	Hazard Ratio (95% Cl)	HR P-value
PreOp Total Music Index*				
Continuous	0.95 (0.92-0.99)	**0.006**	0.95 (0.91-0.99)	**0.016**
**Age 60+**	1.56 (1.07-2.27)	**0.021**	1.56 (0.99-2.44)	0.053
**Gender**				
Male	1.40 (0.93-2.11)	0.111	1.48 (0.91-2.40)	0.114
**Race**				
Black	1.45 (0.95-2.22)	0.088	1.46 (0.85-2.51)	0.175
**Obesity (≥ 30 kg/m2)**	1.37 (0.93-2.00)	0.11	1.18 (0.75-1.85)	0.474
**ECOG**				
≥ 1	1.44 (0.95-2.16)	0.083	1.46 (0.88-2.42)	0.142
**Type of Nephrectomy**				
Partial	0.65 (0.36-1.17)	0.149	0.56 (0.25-1.26)	0.161
**pT-Stage**				
T1	Ref	–	Ref	–
T2	0.91 (0.43-1.94)	0.816	0.74 (0.26-2.12)	0.581
T3	2.05 (1.09-3.87)	**0.027**	1.79 (0.85-3.80)	0.127
T4	2.54 (0.89-7.22)	0.08	2.68 (0.73-9.90)	0.138
**Pathologic N-Stage**				
N1	1.60 (0.88-2.90)	0.122	1.40 (0.62-3.17)	0.424
**Fuhrman Grade 3-4 Disease**				
G3-G4	1.21 (0.78-1.87)	0.401	0.93 (0.56-1.56)	0.788
**ccRCC**	1.02 (0.65-1.60)	0.926	–	–

*[TMA]/height (m2). Clear Cell Renal Carcinoma (ccRCC), Stage, Size, Grade, Necrosis (SSIGN), Eastern Cooperative Oncology Group (ECOG). c-index=0.7302 (full), 0.7222 (ccRCC). Bolded P-Value indicates clinical significance with p<0.05.

## Discussion

In this study, we evaluated the prognostic utility of preoperative linear segmentation for determining survival outcomes in patients with localized RCC undergoing nephrectomy. L3 paraspinal and psoas muscle index muscle index, as measured *via* linear segmentation, was significantly associated with decreased OS as a binary value with the threshold being the median level (Full cohort HR=1.96, 95% CI 1.32-2.90; ccRCC specific HR=1.78, 95% CI 1.08-2.75). Notably, every unit increase in total muscle index is associated with decreased overall mortality (Full cohort HR=0.95, 95% CI 0.92-0.99; ccRCC HR=0.95, 95% CI 0.91-0.99). Importantly, these relationships persisted after controlling for other well-established predictive factors in RCC, such as T-stage, N-stage, and Furhman grade. To our knowledge, this analysis represents the largest study to date evaluating linear segmentation for muscle mass quantification and its prognostic ability in surgical RCC populations. Having a tool present on traditional medical image viewers that can easily and efficiently identify low muscle composition in patients during the preoperative visit has important implications in the management and treatment of RCC patients.

Preoperative sarcopenia has been established as a prognostic factor associated with increased perioperative mortality in surgical patients (5–7), including RCC patients following nephrectomy (8, 10). Skeletal muscle composition can be estimated by mapping the total cross sectional area of skeletal muscle at the level of L3 (12–14); however, the labor, time, and cost extensive nature of this technique limits its clinical use (11). Initial efforts at estimating skeletal muscle mass in various surgical cancer populations by mapping total bilateral psoas or paraspinal muscle groups alone, while more efficient, has provided inconsistent muscle mass quantification and has limited ability to predict outcomes (16, 21–25). Similarly, attempts to simplify body composition *via* digital ruler measurements of the psoas muscles alone have had varying success in different oncologic populations (17, 26).

The linear segmentation technique described in this study and introduced by Avrutin et al. utilizes both the psoas and paraspinal muscle groups, which appear to maintain a stronger correlation with traditional cross sectional area mapping (11). This method utilizes commonly obtained preoperative imaging, can be done quickly during clinic in under a minute, and provides critical information in the assessment of a patient’s perioperative risk. As one of the largest linear segmentation studies, and the largest for RCC, our results provide further support for the implementation of linear segmentation as an inexpensive, expeditious, and clinic friendly alternative for assessing skeletal muscle composition in patients (11, 13, 14). Much like the promising results by Feliciano et al. in a large cohort of colorectal cancer subjects (15), linear segmentation of the bilateral psoas and paraspinal muscle groups measured on axial CT images at the mid L3 vertebra in this large cohort of RCC patients demonstrates prognostic utility and is independently associated with overall survival in patients undergoing surgery for localized disease.

The rationale for why patients with sarcopenia experience shorter overall survival is likely multifactorial. In addition to being associated with traditional aging processes, sarcopenia also results from other factors such as malignancy, comorbidities, therapy regimens, inflammation, and malnutrition (4, 10). Therefore, severity of disease and associated inflammation and malnutrition are likely contributing to the degree of skeletal muscle catabolism (3, 27). Previous studies have examined the influence of malnutrition (i.e. hypoalbuminemia) or inflammation (i.e. C-reactive protein) within nonmetastatic RCC patients, identifying both independent prognostic ability as well as synergism with measured sarcopenia (8, 28).

Thus, quickly identifying patients with low muscle composition can facilitate identification of patients who may benefit from perioperative interventions aimed at building muscle, maximizing nutritional status, and minimizing inflammation. Exercise (i.e. aerobics and resistance) and nutritional supplementation (i.e. anti-inflammatories, amino acids) have exhibited success in reducing perioperative outcomes, cancer mortality, sarcopenia, and frailty due to their propensity to stimulate myoprotein synthesis and anti-inflammatory pathways (3, 15, 29–32). However, a frequent concern regarding these interventions is insufficient time from identification of sarcopenia to treatment or surgical intervention. With nutritional optimization, clinical trials have observed improvements in frailty measures or sarcopenia in as little as 2-8 weeks (33, 34), though sample sizes are limited. Alternatively, in a clinical trial involving frail colorectal cancer patients being assigned multimodal prehabilitation with exercise, nutrition, and psychological intervention, found 4-5 weeks of prehabilitation as insufficient in terms of complications (35). Investigations into prehabilitation programs, their components, and their feasibility are ongoing. Nevertheless, the ability to readily identify patients with a modifiable risk factor such as low muscle mass and encourage prehabilitation is a low risk opportunity to improve patient survival and outcomes (3).

This project is not without its limitations. It is a retrospective, single institutional study, albeit including a large sample size. Reliance in clinical documentation for patient health records can be inconsistent and may have absent variables. Image quality is operator- and patient-dependent and can vary between institutions. Traditional skeletal muscle mapping was not completed on each patient to analyze its correlation with the linear measures, though we believe its correlation in previous studies negates its use in this study. Linear segmentation fails to capture detailed analysis of other muscle or body features such as muscle density, intramuscular fat, or visceral adiposity. This study focused only on patients with localized RCC which may limit the generalizability of the results to metastatic RCC patients. We also looked at non-clear cell RCC as a unit rather than substratifying based on various individual histological subtypes of RCC. However, we believe the principles can be extrapolated to other malignancies, encouraging examination in other populations. Finally, little information exists regarding appropriate muscle index cutoff points. Future research utilizing larger cohorts of various cancer types should focus on identifying TMI cutoffs for optimal risk stratification.

## Conclusions

Linear segmentation of the L3 paraspinal and psoas muscle groups is a practical approach for estimating skeletal muscle composition. In this study, linear segmentation was completed on a large cohort of patients with RCC. As a binary value, lower TMI was associated with decreased overall survival. Similarly, as a continuous measure, increasing muscle index was significantly associated with increased overall survival. These findings support the implementation of linear segmentation into the clinical workflow and its prognostic utility in the preoperative evaluation of patients presenting with nonmetastatic RCC.

## Data availability statement

The datasets presented in this article are not readily available to protect patient health information and ensure maximum anonymity. Requests to access the datasets should be directed to corresponding authors: Viraj A. Master (vmaster@emory.edu) or Benjamin N. Schmeusser (bschmeu@emory.edu).

## Ethics statement

The studies involving human participants were reviewed and approved by Emory University Institutional Review Board. The patients/participants provided their written informed consent to participate in this study.

## Author contributions

BS, EM, AP, DP, AM, MH, MA, RN, SJ, VN, SP, KO, MB and VM contributed to the conception and design of the work. BS, EM, AP, NV, DP, AM, and MH contributed to the acquisition or interpretation of the data for the work. DP was primarily responsible for formal analysis. All authors contributed to the drafting and revision of this manuscript. All authors approved the final form of this manuscript. All authors agree to be accountable for all aspects of this work and its integrity.

## Funding

We gratefully acknowledge support of the John Robinson Family Foundation, Christopher Churchill Foundation, and Cox Immunology Fund.

## Conflict of interest

The authors declare that the research was conducted in the absence of any commercial or financial relationships that could be construed as a potential conflict of interest.

## Publisher’s note

All claims expressed in this article are solely those of the authors and do not necessarily represent those of their affiliated organizations, or those of the publisher, the editors and the reviewers. Any product that may be evaluated in this article, or claim that may be made by its manufacturer, is not guaranteed or endorsed by the publisher.

## Supplementary material

Supplementary Video 1 Real-time linear segmentation *via* the box method example. Measurements were taken of the bilateral psoas and paraspinal muscles at the level of the third lumbar vertebrae.

## Abbreviations

RCC, Renal cell carcinoma; CT, Computed tomography; CT, MRI, Magnetic resonance imaging; L3, Third Lumbar Vertebrae; BMI, Body Mass Index; ECOG, Eastern Cooperative Oncology Group; TMA, Total muscle area; TMI, Total muscle index; OS, Overall survival; ccRCC, Clear cell renal cell carcinoma.

## References

[1] Siegel RL Miller KD Fuchs HE Jemal A. Cancer statistics, 2021. CA Cancer J Clin (2021) 71(1):7–33. doi: 10.3322/caac.21654 33433946

[2] Ljungberg B Albiges L Abu-Ghanem Y Bedke J Capitanio U Dabestani S. European Association of urology guidelines on renal cell carcinoma: The 2022 update. Eur Urol (2022). doi: 10.1016/j.eururo.2022.03.006 35346519

[3] Psutka SP Barocas DA Catto JWF Gore JL Lee CT Morgan TM. Staging the host: Personalizing risk assessment for radical cystectomy patients. Eur Urol Oncol (2018) 1(4):292–304. doi: 10.1016/j.euo.2018.05.010 31100250

[4] Cruz-Jentoft AJ Sayer AA. Sarcopenia. Lancet (2019) 393(10191):2636–46. doi: 10.1016/S0140-6736(19)31138-9 31171417

[5] Sheetz KH Zhao L Holcombe SA Wang SC Reddy RM Lin J. Decreased core muscle size is associated with worse patient survival following esophagectomy for cancer. Dis Esophagus (2013) 26(7):716–22. doi: 10.1111/dote.12020 PMC4171877 23350746

[6] Watanabe S Ishihara H Takagi T Kondo T Ishiyama R Fukuda H. Impact of sarcopenia on post-operative outcomes following nephrectomy and tumor thrombectomy for renal cell carcinoma with inferior vena cava thrombus. Jpn J Clin Oncol (2021) 51(5):819–25. doi: 10.1093/jjco/hyaa275 33558883

[7] Psutka SP Boorjian SA Moynagh MR Schmit GD Frank I Carrasco A. Mortality after radical cystectomy: Impact of obesity versus adiposity after adjusting for skeletal muscle wasting. J Urol (2015) 193(5):1507–13. doi: 10.1016/j.juro.2014.11.088 PMC4556235 25464002

[8] Higgins MI Martini DJ Patil DH Nabavizadeh R Steele S Williams M. Sarcopenia and modified Glasgow prognostic score predict postsurgical outcomes in localized renal cell carcinoma. Cancer (2021) 127(12):1974–83. doi: 10.1002/cncr.33462 33760232

[9] Hu X Liao DW Yang ZQ Yang WX Xiong SC Li X. Sarcopenia predicts prognosis of patients with renal cell carcinoma: A systematic review and meta-analysis. Int Braz J Urol (2020) 46(5):705–15. doi: 10.1590/S1677-5538.IBJU.2019.0636 PMC7822353 32213202

[10] Psutka SP Boorjian SA Moynagh MR Schmit GD Costello BA Thompson RH. Decreased skeletal muscle mass is associated with an increased risk of mortality after radical nephrectomy for localized renal cell cancer. J Urol (2016) 195(2):270–6. doi: 10.1016/j.juro.2015.08.072 26292038

[11] Avrutin E Moisey LL Zhang R Khattab J Todd E Premji T. Clinically practical approach for screening of low muscularity using electronic linear measures on computed tomography images in critically ill patients. JPEN J Parenter Enteral Nutr (2018) 42(5):885–91. doi: 10.1002/jpen.1019 29417591

[12] Kuriyan R. Body composition techniques. Indian J Med Res (2018) 148(5):648–58. doi: 10.4103/ijmr.IJMR_1777_18 PMC6366261 30666990

[13] Mourtzakis M Prado CMM Lieffers JR Reiman T McCargar LJ Baracos VE. A practical and precise approach to quantification of body composition in cancer patients using computed tomography images acquired during routine care. Appl Physiol Nutr Metab (2008) 33(5):997–1006. doi: 10.1139/H08-075 18923576

[14] Steele S Lin F Le TL Medline A Higgins M Sandberg A. Segmentation and linear measurement for body composition analysis using slice-O-Matic and horos. J Vis Exp (2021) 169). doi: 10.3791/61674 33818558

[15] Cespedes Feliciano EM Avrutin E Caan BJ Boroian A Mourtzakis M. Screening for low muscularity in colorectal cancer patients: a valid, clinic-friendly approach that predicts mortality. J Cachexia Sarcopenia Muscle (2018) 9(5):898–908. doi: 10.1002/jcsm.12317 30066490 PMC6204585

[16] Peng PD van Vledder MG Tsai S de Jong MC Makary M Ng J. Sarcopenia negatively impacts short-term outcomes in patients undergoing hepatic resection for colorectal liver metastasis. HPB (2011) 13(7):439–46. doi: 10.1111/j.1477-2574.2011.00301.x PMC3133709 21689226

[17] Jones KI Doleman B Scott S Lund JN Williams JP. Simple psoas cross-sectional area measurement is a quick and easy method to assess sarcopenia and predicts major surgical complications. Colorectal Dis (2015) 17(1):O20–6. doi: 10.1111/codi.12805 25328119

[18] Higgins MI Martini DJ Patil DH Steele S Evans S Petrinec BP. Quantification of body composition in renal cell carcinoma patients: Comparing computed tomography and magnetic resonance imaging measurements. Eur J Radiol (2020) 132:109307. doi: 10.1016/j.ejrad.2020.109307 33010681

[19] Medline A Nabavizadeh R Le TL Patil D Evans S Sandberg A. Magnetic resonance imaging vs. computed tomography image concordance for linear measurements and the quantification of abdominal skeletal muscle. JCSM Clin Rep (2022) 7(1):24–9. doi: 10.1002/crt2.46

[20] Ebbeling L Grabo DJ Shashaty M Dua R Sonnad SS Sims CA. Psoas:lumbar vertebra index: central sarcopenia independently predicts morbidity in elderly trauma patients. Eur J Trauma Emerg Surg (2014) 40(1):57–65. doi: 10.1007/s00068-013-0313-3 26815778 PMC7095912

[21] Peyton CC Heavner MG Rague JT Krane LS Hemal AK. Does sarcopenia impact complications and overall survival in patients undergoing radical nephrectomy for stage III and IV kidney cancer? J Endourol (2016) 30(2):229–36. doi: 10.1089/end.2015.0492 26418428

[22] Smith AB Deal AM Yu H Boyd B Matthews J Wallen EM. Sarcopenia as a predictor of complications and survival following radical cystectomy. J Urol (2014) 191(6):1714–20. doi: 10.1016/j.juro.2013.12.047 24423437

[23] Valero V 3rd Amini N Spolverato G Weiss MJ Hirose K Dagher NN. Sarcopenia adversely impacts postoperative complications following resection or transplantation in patients with primary liver tumors. J Gastrointest Surg (2015) 19(2):272–81. doi: 10.1007/s11605-014-2680-4 PMC4332815 25389056

[24] Taguchi S Akamatsu N Nakagawa T Gonoi W Kanatani A Miyazaki H. Sarcopenia evaluated using the skeletal muscle index is a significant prognostic factor for metastatic urothelial carcinoma. Clin Genitourin Cancer (2016) 14(3):237–43. doi: 10.1016/j.clgc.2015.07.015 26337653

[25] Eo W Kwon J An S Lee S Kim S Nam D. Clinical significance of paraspinal muscle parameters as a prognostic factor for survival in gastric cancer patients who underwent curative surgical resection. J Cancer (2020) 11(19):5792–801. doi: 10.7150/jca.46637 PMC7477437 32913472

[26] Rutten IJG Ubachs J Kruitwagen RFPM Beets-Tan RGH Olde Damink SWM Van Gorp T. Psoas muscle area is not representative of total skeletal muscle area in the assessment of sarcopenia in ovarian cancer. J Cachexia Sarcopenia Muscle (2017) 8(4):630–8. doi: 10.1002/jcsm.12180 PMC5566632 28513088

[27] Feliciano EMC Kroenke CH Meyerhardt JA Prado CM Bradshaw PT Kwan ML. Association of systemic inflammation and sarcopenia with survival in nonmetastatic colorectal cancer: Results from the c SCANS study. JAMA Oncol (2017) 3(12):e172319. doi: 10.1001/jamaoncol.2017.2319 28796857 PMC5824285

[28] Midenberg E Higgins MI Schmeusser BN Patil DH Zaldumbide J Martini DJ. Prognostic value of sarcopenia and albumin in the surgical management of localized renal cell carcinoma. Urol Oncol: Semin Orig Investigations (2022). doi: 10.1016/j.urolonc.2022.09.020 36280529

[29] Nascimento W Ferrari G Martins CB Rey-Lopez JP Izquierdo M Lee DH. Muscle-strengthening activities and cancer incidence and mortality: a systematic review and meta-analysis of observational studies. Int J Behav Nutr Phys Act (2021) 18(1):69. doi: 10.1186/s12966-021-01142-7 34051796 PMC8164763

[30] Soares JDP Howell SL Teixeira FJ Pimentel GD. Dietary amino acids and immunonutrition supplementation in cancer-induced skeletal muscle mass depletion: A mini-review. Curr Pharm Des (2020) 26(9):970–8. doi: 10.2174/1381612826666200218100420 32067606

[31] Pamoukdjian F Bouillet T Lévy V Soussan M Zelek L Paillaud E. Prevalence and predictive value of pre-therapeutic sarcopenia in cancer patients: A systematic review. Clin Nutr (2018) 37(4):1101–13. doi: 10.1016/j.clnu.2017.07.010 28734552

[32] Hill A Arora RC Engelman DT Stoppe C. Preoperative treatment of malnutrition and sarcopenia in cardiac surgery: New frontiers. Crit Care Clin (2020) 36(4):593–616. doi: 10.1016/j.ccc.2020.06.002 32892816

[33] Ritch CR Cookson MS Clark PE Chang SS Fakhoury K Ralls V. Perioperative oral nutrition supplementation reduces prevalence of sarcopenia following radical cystectomy: Results of a prospective randomized controlled trial. J Urol (2019) 201(3):470–7. doi: 10.1016/j.juro.2018.10.010 30359680

[34] Yamamoto K Nagatsuma Y Fukuda Y Hirao M Nishikawa K Miyamoto A. Effectiveness of a preoperative exercise and nutritional support program for elderly sarcopenic patients with gastric cancer. Gastric Cancer (2017) 20(5):913–8. doi: 10.1007/s10120-016-0683-4 28032232

[35] Carli F Bousquet-Dion G Awasthi R Elsherbini N Liberman S Boutros M. Effect of multimodal prehabilitation vs postoperative rehabilitation on 30-day postoperative complications for frail patients undergoing resection of colorectal cancer: A randomized clinical trial. JAMA Surg (2020) 155(3):233–42. doi: 10.1001/jamasurg.2019.5474 PMC6990653 31968063

